# Cumulative non-high-density lipoprotein cholesterol exposure and residual cardiovascular mortality risk in patients with peripheral artery disease

**DOI:** 10.1186/s12944-026-03022-0

**Published:** 2026-08-05

**Authors:** Yujia Zhang, Zhoudong Jing, Zhiqiang Liu, Angel Lai, GuangMing Tan, Bryan P. Yan

**Affiliations:** 1 Division of Cardiology, Department of Medicine and Therapeutics, Faculty of Medicine, The Chinese University of Hong Kong, Shatin, NT Hong Kong; 2 Department of Biostatistics, City University of Hong Kong, 83 Tat Chee Avenue, Kowloon Tong, Kowloon, Hong Kong

**Keywords:** Peripheral arterial disease, Cardiovascular risk, Cholesterol, Longitudinal studies

## Abstract

**Background:**

Individuals with peripheral artery disease (PAD) face substantial residual cardiovascular risk despite lipid-lowering therapy. Non-high-density lipoprotein cholesterol (non-HDL-C) has been recognized as a more comprehensive marker of cardiovascular risk than low-density lipoprotein cholesterol (LDL-C), but its prognostic value in PAD and the relevance of cumulative exposure remain unclear. This study investigated whether early cumulative non-HDL-C exposure is associated with cardiovascular death (CV death) in PAD and whether the same association is observed among individuals whose LDL-C is already well controlled.

**Methods:**

A territory-wide PAD cohort in Hong Kong was analyzed. Early cumulative non-HDL-C and LDL-C exposures were defined as the lipid burden during the first 2 years after PAD diagnosis and were estimated using spline models. In the overall cohort, Fine-Gray competing-risk models were applied to assess the associations between tertiles of cumulative non-HDL-C exposure and CV death, with non-cardiovascular mortality treated as a competing event. Discordance analyses compared CV death risk across nine strata defined by cross-classified tertiles of cumulative LDL-C and non-HDL-C, with the low-LDL-C/low-non-HDL-C stratum as the reference. In the well-controlled LDL-C subgroup, the same competing risk framework was used to assess the residual risk of CV death across cumulative non-HDL-C categories.

**Results:**

Among 3,847 patients, 637 CV deaths occurred during a mean follow-up of 7.30 ± 3.92 years. Higher cumulative non-HDL-C exposure was associated with a higher risk of CV death (subdistribution hazard ratio [SHR], 1.36; 95% confidence interval [CI], 1.11–1.65; *P* = 0.002). Notably, patients with high non-HDL-C despite low LDL-C showed higher CV death risk (SHR, 2.00; 95% CI, 1.12–3.57; *P* = 0.018). In the 516 individuals with well-controlled LDL-C, higher cumulative exposure of non-HDL-C remained significantly related to CV death risk (SHR, 2.68; 95% CI, 1.39–5.16; *P* = 0.003).

**Conclusions:**

Higher early cumulative non-HDL-C exposure was associated with a higher risk of CV death in PAD, including individuals who had attained the LDL-C target. Incorporating non-HDL-C into routine lipid follow-up may help identify residual cardiovascular risk, improve risk stratification, and support secondary prevention in patients with PAD, thereby addressing the long-term cardiovascular burden in this population.

**Supplementary Information:**

The online version contains supplementary material available at https://doi.org/10.1186/s12944-026-03022-0.

## Introduction

Peripheral artery disease (PAD) is a common manifestation of systemic atherosclerosis, affecting over 230 million people globally and contributing substantially to cardiovascular morbidity and mortality [1–3]. Despite advances in medical therapy, the residual cardiovascular risk remains high in PAD, emphasizing the need for improved lipid management [4–7]. Low-density lipoprotein cholesterol (LDL-C) has been widely regarded as the primary therapeutic target in cardiovascular prevention [5, 8]. However, growing evidence indicates that non-high-density lipoprotein cholesterol (non-HDL-C), which includes LDL-C, very-low-density lipoprotein cholesterol (VLDL-C), intermediate-density lipoprotein cholesterol (IDL-C), and lipoprotein(a), may offer superior prognostic value by capturing the atherogenic lipoprotein burden [9–12]. Notably, elevated non-HDL-C concentrations were related to higher cardiovascular risk among individuals with ischemic heart disease receiving statin therapy [13], even among those with well-controlled LDL-C, defined as LDL-C ≤ 1.8 mmol/L in that study [14].

Diabetes, insulin resistance, and related metabolic disturbances are common in PAD and promote an atherogenic dyslipidemia profile dominated by triglyceride-rich/remnant lipoproteins and small dense low-density lipoprotein particles [15]. In this setting, LDL-C may underestimate the total atherogenic particle burden, whereas non-HDL-C may better capture triglyceride-rich and remnant cholesterol. Consistent with this, large cohort and genetic studies have indicated that elevated remnant cholesterol accounts for a substantial proportion of the apparent causal link between LDL-C and PAD [16, 17], underscoring that atherogenic components beyond LDL-C are clinically relevant in this population.

The American College of Cardiology guidelines recognize non-HDL-C as a secondary treatment target in high-risk groups, including patients with established atherosclerotic cardiovascular disease [18]. Moreover, longitudinal cohort studies have shown that cumulative lipid exposure over time is more strongly associated with outcomes than baseline measurement alone [19, 20]. More specifically, early attainment and sustained maintenance of lower non-HDL-C concentrations after myocardial infarction were linked to a lower risk of cardiovascular outcomes [21]. Cesaro et al. reported lower visit-to-visit LDL-C variability with proprotein convertase subtilisin/kexin type 9 (PCSK9) inhibitors and inclisiran than with standard lipid-lowering therapy, while lower variability was associated with a more favorable cardiovascular risk profile [22]. Because lipid variability and cumulative lipid exposure both reflect longitudinal lipid patterns, longitudinal lipid measurements may provide clinically important information on cardiovascular risk beyond single lipid measurements. However, PAD-specific evidence remains limited. Ding et al. examined non-HDL-C in relation to prevalent PAD in a cross-sectional study but did not address cardiovascular outcomes after PAD diagnosis [23]. Hageman et al. evaluated a single baseline non-HDL-C measurement in relation to limb and composite cardiovascular outcomes but did not assess whether long-term non-HDL-C burden was related to outcomes [24].

Given that cumulative lipid exposure reflects longitudinal lipid burden more accurately than single measurements and considering the atherogenic lipid characteristics of PAD, it was hypothesized that higher early cumulative non-HDL-C exposure would be associated with residual cardiovascular risk in PAD. Using data from the Clinical Data Analysis and Reporting System (CDARS), this study examined the association between early cumulative non-HDL-C exposure after PAD diagnosis and cardiovascular death and further evaluated this association among those with well-controlled LDL-C levels. Unlike previous studies based on single-time-point lipid measurements, this study assessed longitudinal cumulative non-HDL-C exposure in a territory-wide real-world PAD cohort and specifically addressed residual cardiovascular risk despite LDL-C target attainment.

## Methods

The Joint Chinese University of Hong Kong-New Territories East Cluster Clinical Research Ethics Committee approved the study (Ref. No. 2018.304). All procedures complied with the Declaration of Helsinki. As this analysis used fully anonymized registry records, the committee granted a waiver of written informed consent.

### Data source

Anonymized patient-level records were retrieved through the CDARS, a territory-wide electronic health record database covering nearly 80% of Hong Kong residents. Details of CDARS are reported in an earlier publication [25].

### Study design and participants

First analysis: Overall PAD cohort. The initial cohort included 27,641 individuals whose first recorded PAD diagnosis occurred between July 15, 2003, and December 31, 2023, as identified using the International Classification of Diseases, Ninth Revision, Clinical Modification (ICD-9-CM) codes [26]. To assess the cumulative lipid exposure, a fixed 2-year exposure window after the index PAD diagnosis was defined for all patients. Because lipid tests were performed at irregular intervals in routine care, eligible individuals were required to have at least one complete lipid profile (total cholesterol [TC], high-density lipoprotein cholesterol [HDL-C], and LDL-C) at baseline and at least one profile within the 6 months preceding the start of follow-up (i.e., between 18 and 24 months after diagnosis), so that lipid levels were observed near both the beginning and end of the exposure window. Follow-up commenced two years post-PAD diagnosis to evaluate the impact of early-stage lipid control on long-term prognosis. From the initial cohort, patients were sequentially excluded for age < 18 years (*n* = 24), follow-up < 2 years (*n* = 11,218), incomplete lipid profiles at baseline and/or within 6 months preceding the start of follow-up (*n* = 12,034), and baseline malignancy, hematologic disease, or end-stage hepatic or renal dysfunction (*n* = 518). The final analytical cohort comprised 3,847 individuals. Supplementary Figure S1 shows the participant flowchart.

Secondary analysis: Well-controlled LDL-C subgroup. A secondary analysis was conducted to assess the relationship between cumulative non-HDL-C exposure and cardiovascular death (CV death) in patients who achieved recommended LDL-C control. This subgroup was derived from the primary analytical cohort, as described above. Based on the European Society of Cardiology guideline-recommended LDL-C target for very-high-risk individuals [5], well-controlled LDL-C was defined in this analysis as at least two LDL-C measurements within the 6 months before the start of follow-up, both < 1.4 mmol/L and showing a ≥ 50% reduction from baseline. In total, 516 individuals met the criteria for well-controlled LDL-C levels (Supplementary Figure S1).

### Risk factor assessment and other measurements

Age and sex were ascertained at the time of PAD diagnosis. The analysis used ICD-9-CM codes to ascertain comorbidities that were present before PAD diagnosis, including hypertension, chronic kidney disease (CKD), and diabetes, with cardiovascular comorbidities defined as the presence of at least one of coronary artery disease (CAD), stroke, heart failure, or arrhythmias. PAD severity was categorized as asymptomatic, intermittent claudication (IC), or critical limb ischemia (CLI) (Supplementary Table S1). Laboratory parameters were defined using the values recorded closest to the index PAD diagnosis within a window from 6 months before to 1 week after the diagnosis. Non-HDL-C was derived by subtracting HDL-C from TC [27]. The estimated glomerular filtration rate (eGFR) was derived from the Chronic Kidney Disease Epidemiology Collaboration Eq. [28]. Medication use at baseline, including antiplatelet agents, angiotensin-converting enzyme inhibitors (ACEIs) or angiotensin receptor blockers (ARBs), and lipid-lowering drugs, was retrieved from prescription records. Patients were considered to be receiving a given agent at baseline if at least one prescription for that agent was issued between 6 months before and 1 week after the index PAD diagnosis. In addition, statin intensity and patterns of lipid-lowering therapy at baseline and during the 2-year exposure window were summarized; detailed definitions of statin intensity are provided in Supplementary Table S2. During the 2-year exposure window, adherence was assessed among patients receiving any lipid-lowering medication at baseline using prescription coverage, expressed as the proportion of days covered (PDC) over the 2-year period; PDC ≥ 80% denoted adherence [29].

### Endpoint

CV death, comprising cardiac and cerebrovascular mortality, was the primary endpoint and was identified using codes from the Tenth Revision of the ICD (ICD-10). All-cause mortality was assessed as the secondary endpoint. Vital status data came from the CDARS database.

### Estimation of cumulative lipid exposure

Within the 2-year exposure window after PAD diagnosis, the analysis included all available LDL-C and non-HDL-C measurements without imputing missing lipid values. Based on approaches adopted in previous studies, a polynomial spline model with mixed effects estimated subject-specific trajectories for both LDL-C and non-HDL-C in each individual. The model accommodates nonlinear lipid trajectories and handles sparse or irregular measurements by borrowing the strength of population-level trends through random effects. The area under each fitted trajectory over 0–2 years was calculated as cumulative exposure (AUC; mmol/L × year). For ease of interpretation, the AUC was divided by two years to obtain a time-averaged lipid burden (mmol/L), representing the average level of the corresponding lipid measure over the 2-year period [30].

### Statistical analysis

Baseline characteristics were summarized and compared among participants classified into tertiles of cumulative non-HDL-C exposure. Continuous variables were analyzed using one-way analysis of variance (ANOVA) when their distributions were approximately normal and the Kruskal-Wallis test otherwise. Categorical variables were compared using the chi-square test or Fisher’s exact test, as appropriate. Continuous data are presented as mean ± standard deviation (SD) or median (interquartile range [IQR]), whereas categorical data are reported as n (%). Key covariates including age, sex, comorbidities, and medications had no missing values; eGFR was missing in only 6 patients (0.16%) and was handled using a random-forest implementation of multiple imputation by chained equations (MICE) (Supplementary Table S3).

Associations between cumulative lipid burden and cardiovascular death were assessed using Fine-Gray models to estimate subdistribution hazard ratios (SHRs), treating non-cardiovascular death as a competing risk [31]. A Cox proportional hazards model was fitted for all-cause mortality to estimate hazard ratios (HRs). Multivariable adjustment included age; sex; hypertension; diabetes; eGFR; cardiovascular comorbidities; PAD severity; antiplatelet, ACEIs/ARBs, and statin therapy. Harrell’s C-statistic, integrated discrimination improvement (IDI) and net reclassification improvement (NRI) were calculated with 95% confidence intervals (CIs) to evaluate discrimination and reclassification when comparing models incorporating cumulative non-HDL-C with those incorporating cumulative or baseline LDL-C [32].

The discordance analysis categorized each participant separately according to cumulative LDL-C and cumulative non-HDL-C tertiles. Tertile 1, Tertile 2, and Tertile 3 represented low, intermediate, and high exposure, respectively. Cross-classification in a 3 × 3 matrix generated nine strata. The stratum with both LDL-C and non-HDL-C in Tertile 1 served as the reference. Strata in which both lipid markers were in the same tertile were considered concordant, whereas those with different LDL-C and non-HDL-C tertiles were considered discordant. For the well-controlled LDL-C subgroup, the analysis further grouped cumulative non-HDL-C exposure into < 30th, 30th–90th, and ≥ 90th percentiles and assessed residual risk using Fine-Gray subdistribution hazard models. Individual lipid trajectories during the two-year exposure window were visualized, and the distribution of non-HDL-C exposure within each category was summarized descriptively to aid clinical interpretation.

Four sensitivity analyses examined whether the observed associations of cumulative lipid burden with mortality were robust: (a) extending the exposure period to 3 years for more measurements, (b) shortening the exposure window to 1 year to focus on the very early post-diagnosis period, (c) additionally adjusting for changes in statin intensity from baseline to the end of the 2-year exposure assessment period, and (d) excluding patients with baseline cardiovascular comorbidities to reduce potential confounding. To validate the mortality associations for discordant lipid patterns, two complementary analyses were performed: (a) requiring ≥ 3 lipid measurements to calculate cumulative exposure; (b) using a minimally adjusted model (age, sex, diabetes, PAD severity, cardiovascular comorbidities, statin use). In the well-controlled LDL-C subgroup, the analyses were repeated using an alternative definition in which the most recent LDL-C level before follow-up was < 1.4 mmol/L. All statistical procedures were implemented in R, version 4.4.2.

## Results

### Baseline characteristics across cumulative non-HDL-C tertiles

The primary cohort included 3,847 patients with PAD (mean age 70.03 ± 11.30 years; 63.0% male). Over a mean follow-up of 7.30 ± 3.92 years, 2,390 deaths (62.1%) occurred, including 637 CV deaths (16.6%). Within the 2-year exposure window, patients had a mean of 5.13 ± 2.18 non-HDL-C measurements, with median inter-test intervals typically 3–7 months (Supplementary Figures S2-S3). Table 1 summarizes baseline characteristics across cumulative non-HDL-C tertiles. Participants in the higher tertiles were younger, less often male, and had higher baseline TC, LDL-C, triglyceride (TG), and glycated hemoglobin (HbA1c) levels (all *P* < 0.001). Conversely, the prevalence of comorbidities, including hypertension, cardiovascular disease, and diabetes, declined with increasing non-HDL-C tertiles, and comparable patterns were observed when stratified by cumulative LDL-C levels (Supplementary Table S4). In addition, the majority of patients (66.5%) were receiving statins, including 5.5% who were on high-intensity statin therapy, whereas other lipid-lowering agents were prescribed to only approximately 5.1% of patients. Notably, PCSK9 inhibitors were used in only 0.2% of the patients (Table 1, Supplementary Table S5). During the 2-year exposure window, statin use increased further to 81.4%, and 11.6% of the patients received high-intensity statin therapy at least once, while the use of other lipid-lowering agents remained low (Supplementary Table S6). Among patients receiving lipid-lowering therapy at baseline, the median 2-year PDC was 96.4% (IQR, 89.1%–99.9%), and 85.7% achieved PDC ≥ 80%, indicating generally sustained use of lipid-lowering therapy over the exposure window (Supplementary Figure S4).

**Table 1:** Baseline characteristics of participants according to categories of cumulative exposure of non-HDL-C

Characteristics	Tertile 1 *N* = 1,283	Tertile 2 *N* = 1,282	Tertile 3 *N* = 1,282	Total *N* = 3,847	*P* value
Non-HDL-C (Cumulative Exposure), mmol/L	2.33 [2.10, 2.49]	2.92 [2.78, 3.06]	3.65 [3.40, 4.02]	2.92 [2.49, 3.40]	< 0.001
Non-HDL-C (Baseline), mmol/L	2.26 [1.93, 2.60]	3.01 [2.66, 3.46]	4.01 [3.44, 4.71]	3.00 [2.40, 3.78]	< 0.001
Demographic					
Male Sex, %	872 (68.0%)	781 (60.9%)	769 (60.0%)	2422 (63.0%)	< 0.001
Age, years	71.69 ± 10.95	70.35 ± 11.19	68.04 ± 11.48	70.03 ± 11.30	< 0.001
Comorbidities					
Hypertension, %	784 (61.1%)	730 (56.9%)	645 (50.3%)	2159 (56.1%)	< 0.001
Cardiovascular disease, %	840 (65.5%)	691 (53.9%)	531 (41.4%)	2062 (53.6%)	< 0.001
CKD, %	286 (22.3%)	271 (21.1%)	274 (21.4%)	831 (21.6%)	0.755
Diabetes, %	838 (65.3%)	794 (61.9%)	746 (58.2%)	2378 (61.8%)	0.001
Laboratory					
Total Cholesterol, mmol/L	3.46 [3.06, 3.90]	4.20 [3.80, 4.73]	5.19 [4.56, 5.90]	4.20 [3.57, 5.00]	< 0.001
High-density lipoprotein, mmol/L	1.15 [0.93, 1.42]	1.11 [0.92, 1.39]	1.10 [0.92, 1.34]	1.12 [0.92, 1.38]	0.005
Low-density lipoprotein, mmol/L	1.72 [1.40, 2.06]	2.38 [2.00, 2.77]	3.18 [2.58, 3.81]	2.32 [1.79, 3.05]	< 0.001
Triglycerides, mmol/L	1.10 [0.80, 1.46]	1.31 [0.98, 1.77]	1.57 [1.15, 2.30]	1.30 [0.95, 1.82]	< 0.001
HbA1c, %	6.73 [6.10, 7.60]	7.00 [6.20, 8.10]	7.00 [6.20, 8.20]	6.90 [6.20, 7.92]	< 0.001
eGFR, mL/min/1.73 m²	57.40 [33.72, 79.76]	58.60 [34.54, 81.60]	58.12 [32.13, 82.58]	58.19 [33.17, 81.10]	0.6
Medications					
ACEIs/ARBs	848 (66.1%)	800 (62.4%)	685 (53.4%)	2333 (60.6%)	< 0.001
Beta-blockers	604 (47.1%)	525 (41.0%)	475 (37.1%)	1604 (41.7%)	< 0.001
Antiplatelet	1016 (79.2%)	969 (75.6%)	860 (67.1%)	2845 (74.0%)	< 0.001
Antidiabetic drugs	475 (37.0%)	459 (35.8%)	411 (32.1%)	1345 (35.0%)	0.023
Lipid-lowering medications					
Statin	1016 (79.2%)	878 (68.5%)	666 (52.0%)	2560 (66.5%)	< 0.001
Non-statin lipid-lowering medications	43 (3.4%)	57 (4.4%)	98 (7.6%)	198 (5.1%)	< 0.001
Fibrate	14 (1.1%)	47 (3.7%)	81 (6.3%)	142 (3.7%)	< 0.001
Ezetimibe	22 (1.7%)	6 (0.5%)	11 (0.9%)	39 (1.0%)	0.006
PCSK9 inhibitor	3 (0.2%)	3 (0.2%)	3 (0.2%)	9 (0.2%)	> 0.99
Others	4 (0.3%)	1 (0.1%)	3 (0.2%)	8 (0.2%)	0.417

Values are presented as mean ± SD, median (IQR), or n (%), as appropriate. Analyses were based on cumulative non-HDL-C levels during the early exposure period. Patients were considered to be receiving a given agent if at least one prescription for that agent was issued during the baseline period from 6 months before to 1 week after the index PAD diagnosis

*Non-HDL-C* non-high-density lipoprotein cholesterol, *CKD* chronic kidney disease, *HbA1c* glycated hemoglobin, *eGFR* estimated glomerular filtration rate, *ACEIs* angiotensin-converting enzyme inhibitors, *ARBs* angiotensin receptor blockers

### Associations of baseline and cumulative lipid burden with mortality

**Fig. 1:**
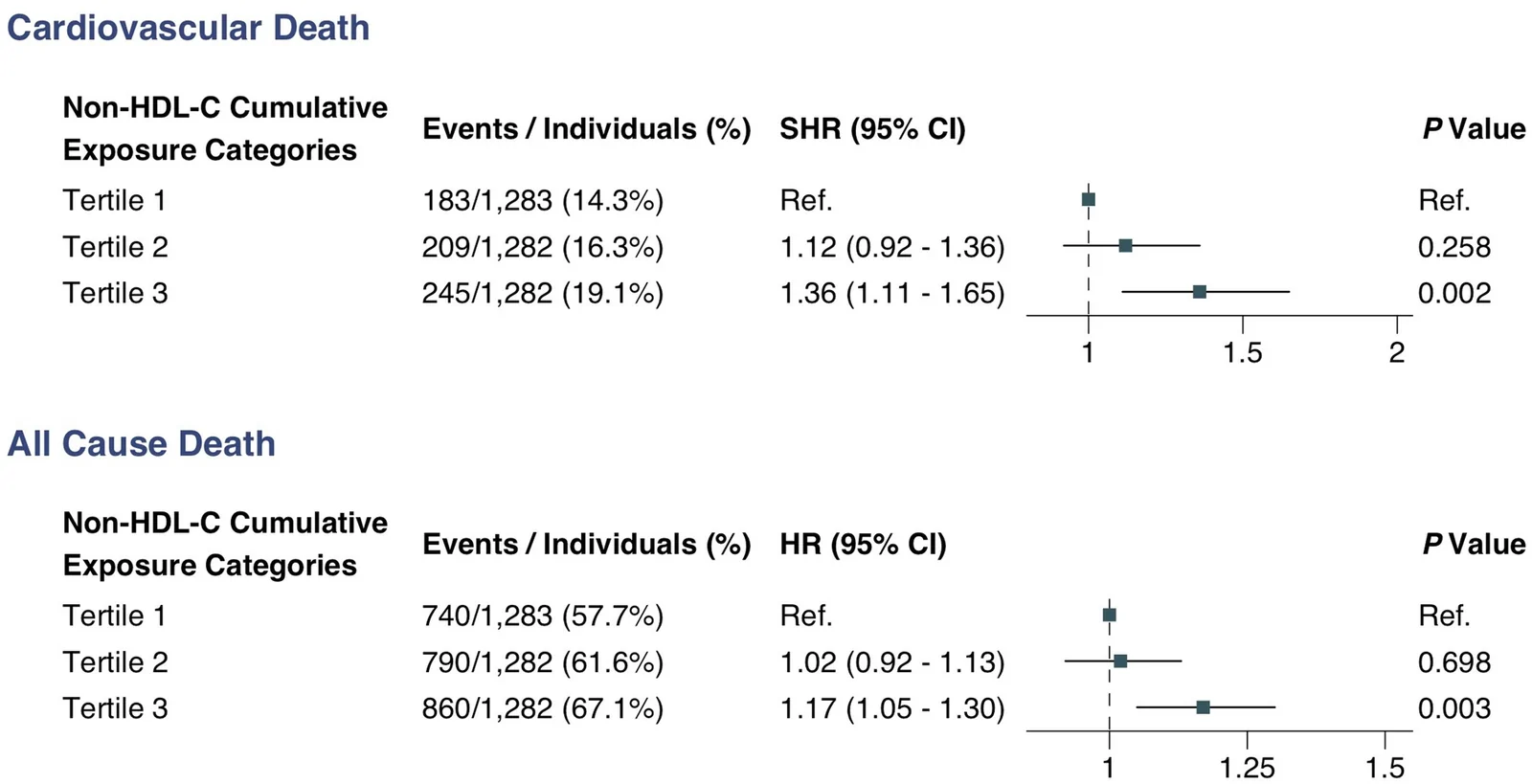
Associations Between Early Cumulative Exposure to Non-HDL-C and Risk of All-Cause and Cardiovascular Death. Note: Cumulative non-HDL-C was categorized into tertiles (T1-T3), with T1 as the reference. Higher cumulative exposure was associated with increased risks of both outcomes, shown as adjusted HRs and SHRs. Models were adjusted for age, sex, hypertension, diabetes, eGFR, cardiovascular comorbidities, PAD severity, and use of antiplatelet agents, ACEIs/ARBs, and statins. HR hazard ratio, SHR subdistribution hazard ratio, CI confidence interval, eGFR estimated glomerular filtration rate, PAD peripheral artery disease, ACEIs angiotensin-converting enzyme inhibitors, ARBs angiotensin receptor blockers

During the early phase following PAD diagnosis, the highest tertile of cumulative non-HDL-C burden was related to a greater risk of cardiovascular mortality (SHR, 1.36; 95% CI, 1.11–1.65; *P* = 0.002). A similar pattern was observed for cumulative LDL-C (SHR, 1.23; 95% CI, 1.01–1.49; *P* = 0.041). For all-cause mortality, the HRs for Tertile 3 (highest level) versus Tertile 1 (lowest level) were 1.17 (95% CI, 1.05–1.30; *P* = 0.003) for cumulative non-HDL-C and 1.21 (95% CI, 1.09–1.35; *P* = 0.001) for cumulative LDL-C (Fig. 1; Supplementary Figure S5). Across tertiles of cumulative non-HDL-C, the CV death rates were 25.0, 25.8 and 28.8 per 1,000 person-years in tertiles 1–3, with corresponding all-cause death rates of 97.5, 101.1 and 101.2 per 1,000 person-years. Similar incidence patterns were observed for cumulative LDL-C levels (Supplementary Table S7). Kaplan-Meier curves showed poorer survival in Tertile 3 for both endpoints (Supplementary Figures S6A-S6B). Additionally, at baseline, CV death risk was higher in the highest LDL-C category (SHR, 1.26; 95% CI, 1.01–1.58; *P* = 0.042) and the highest non-HDL-C category (SHR, 1.35; 95% CI, 1.08–1.69; *P* = 0.008). Neither baseline measure was associated with all-cause mortality (Supplementary Figures S7-S8). Model performance analyses showed that cumulative lipid exposure improved the model discrimination for CV death compared to baseline measures. Compared with the model including cumulative burden of LDL-C, the model incorporating cumulative non-HDL-C showed modest improvements with ΔC = 0.0015 (95% CI, 0.0003–0.0028), IDI = 0.0017 (95% CI, 0.0010–0.0024), and NRI = 13.3% (95% CI, 6.8–19.8%) (Supplementary Table S8).

### Discordance analysis

**Fig. 2:**
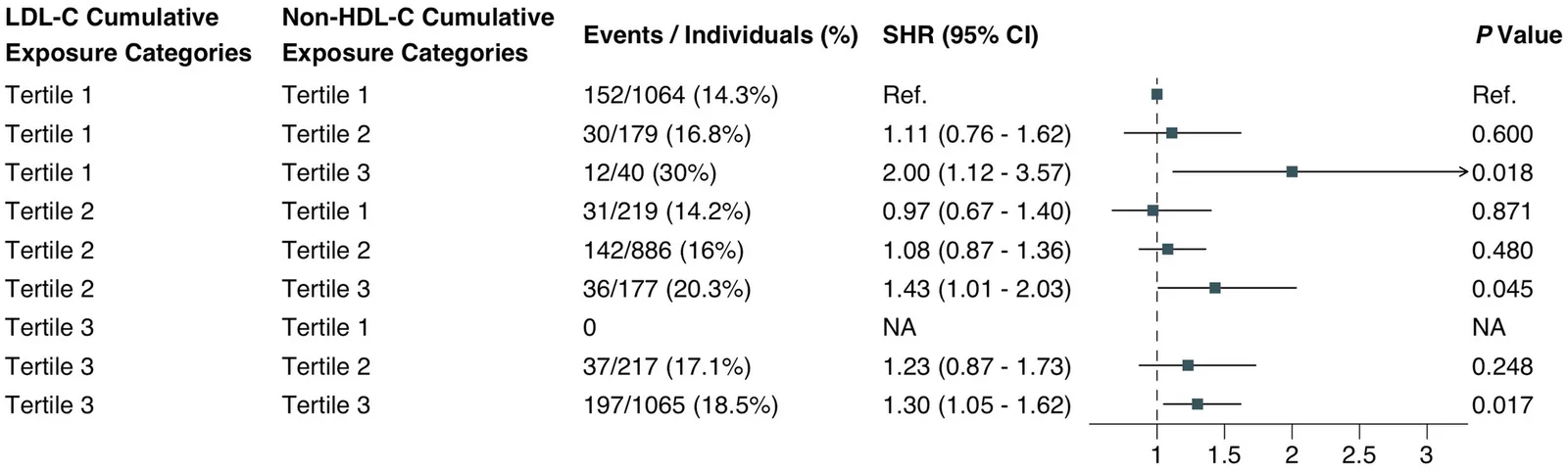
Hazard Ratios for Cardiovascular Death Across Concordant and Discordant Categories of Early Cumulative LDL-C and Non-HDL-C. Note: Participants were categorized into nine strata by cross-classifying tertiles of cumulative LDL-C and cumulative non-HDL-C during the 2-year exposure window. Tertile 1, Tertile 2, and Tertile 3 indicate low, intermediate, and high cumulative exposure, respectively. The low-LDL-C/low-non-HDL-C stratum (Tertile 1/Tertile 1) was used as the reference. Strata in which LDL-C and non-HDL-C were in the same tertile were considered concordant, whereas strata with different LDL-C and non-HDL-C tertiles were considered discordant. Models were adjusted for age, sex, hypertension, diabetes, eGFR, cardiovascular comorbidities, PAD severity, and use of antiplatelet agents, ACEIs/ARBs, and statins. SHR, subdistribution hazard ratio; CI, confidence interval; eGFR, estimated glomerular filtration rate; PAD, peripheral artery disease; ACEIs, angiotensin-converting enzyme inhibitors; ARBs, angiotensin receptor blockers

In discordance analyses, compared with patients in the lowest tertiles of both cumulative non-HDL-C and LDL-C exposure, those in the highest tertiles for both markers exhibited consistently higher risks of cardiovascular and all-cause mortality (SHR, 1.30; 95% CI, 1.05–1.62; *P* = 0.017 and HR, 1.18; 95% CI, 1.05–1.32; *P* = 0.006, respectively) (Fig. 2; Supplementary Figure S9). Among discordant subgroups, individuals with high cumulative non-HDL-C but low LDL-C burden had the highest risk of CV death (SHR, 2.00; 95% CI, 1.12–3.57; *P* = 0.018), followed by those with high non-HDL-C and intermediate LDL-C (SHR, 1.43; 95% CI, 1.01–2.03; *P* = 0.045). These associations were not significant when extended to all-cause mortality (Supplementary Figure S9).

### Cardiovascular risk in individuals withwell-controlled LDL-C

**Fig. 3:**
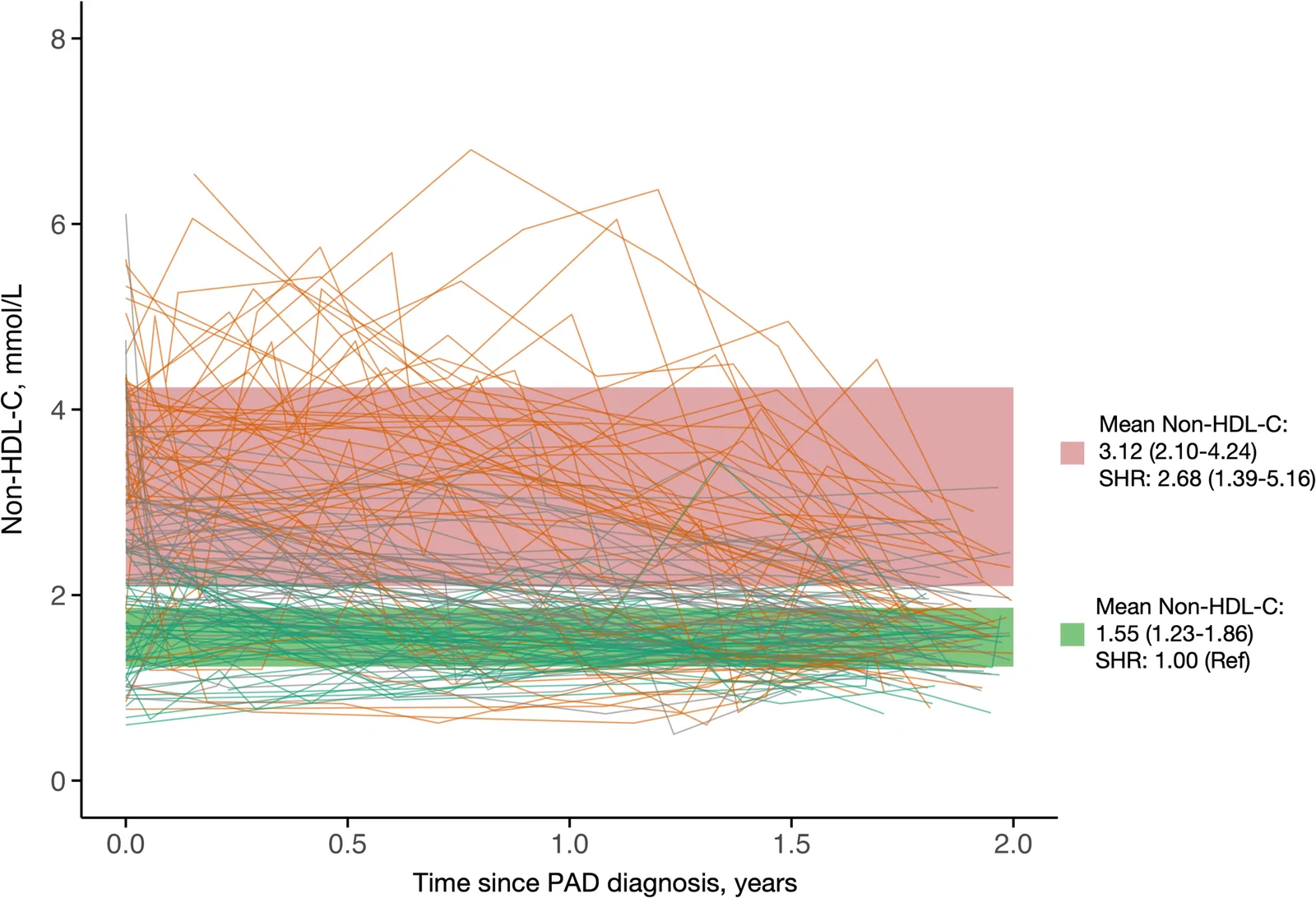
Individual Non-HDL-C Trajectories According to Cumulative Non-HDL-C Exposure Categories in Patients with Well-Controlled LDL-C. Note: Cumulative non-HDL-C exposure was categorized into < 30th and ≥ 90th percentiles. Individual trajectories over the 2-year exposure window are shown, together with the mean time-averaged non-HDL-C burden for each category. The < 30th percentile group served as the reference. The higher non-HDL-C group demonstrated a significantly increased risk of CV death compared with the reference group. SHR, subdistribution hazard ratio; CI, confidence interval

In a secondary cohort of 516 individuals meeting the prespecified LDL-C control criteria, the highest cumulative non-HDL-C category (mean, 3.12 mmol/L) had a higher risk of CV death than the lowest category (mean, 1.55 mmol/L), with an SHR of 2.68 (95% CI, 1.39–5.16; *P* = 0.003) (Supplementary Figure S10). Individual non-HDL-C trajectories over the 2-year exposure window are shown in Fig. 3.

### Sensitivity analysis

Extending or shortening the exposure window and excluding participants with baseline cardiovascular comorbidities yielded consistent associations of both cumulative lipid measures with cardiovascular and all-cause mortality (Supplementary Figures S11A-S13B). Additional adjustment for changes in statin intensity produced a similar estimate for cumulative non-HDL-C and CV death (Supplementary Figure S14). In analyses requiring ≥ 3 lipid measurements and in a minimally adjusted model, the elevated CV death risk in the discordant lipid pattern was confirmed, with SHR estimates ranging from 1.91 to 2.22 (all *P* < 0.05), further supporting the robustness of the observed associations (Supplementary Figures S15A–S16B). Under the alternative LDL-C-control definition based on the latest pre-follow-up value < 1.4 mmol/L, cumulative non-HDL-C remained significantly related to CV death (Supplementary Figure S17).

## Discussion

This study demonstrated that higher early cumulative non-HDL-C burden after PAD diagnosis was related to higher cardiovascular and all-cause mortality risk. Importantly, this association with CV death was also observed among individuals who met the LDL-C target, indicating that non-HDL-C may complement LDL-C in cardiovascular risk assessment and secondary prevention in PAD.

Although non-HDL-C is widely acknowledged as a comprehensive marker of cardiovascular risk in the general population [33–36], evidence in PAD cohorts remains limited. Ding et al. and Hageman et al. examined its relationship with PAD or limb outcomes but did not address the impact of long-term lipid burden [23, 24]. As non-HDL-C levels fluctuate with lifestyle and treatment, a single baseline measurement may not capture sustained burden [19]. Cumulative exposure provides a closer representation of the atherogenic burden experienced during routine clinical care. This may be particularly relevant in PAD, because individuals with PAD often have multiple chronic comorbidities, and their lipid levels may vary with medication changes or interactions, treatment adherence, concomitant conditions, and metabolic status. Accordingly, compared with an isolated baseline value, cumulative non-HDL-C may provide a more integrated measure of lipid burden under the combined influence of these factors. Elevated cumulative non-HDL-C burden has been linked to subsequent atherosclerotic cardiovascular disease (ASCVD) events [20, 30] and higher mortality risk [37], further supporting the present findings. In addition, a large European survey reported that individuals with coronary heart disease (CHD) and concomitant PAD had a less favorable cardiovascular risk profile, including more frequent smoking and higher prevalences of diabetes, obesity, hypertriglyceridemia, and low HDL-C, as well as substantially impaired quality of life, than those without PAD [38]. These observations suggest that patients with PAD may represent a group with more advanced atherosclerosis and particularly high residual cardiovascular risk, providing external context for these findings and underscoring the importance of lipid assessment beyond LDL-C alone in this population.

A notable finding was that the discordant pattern of low cumulative LDL-C and high cumulative non-HDL-C showed the highest association with CV death. This pattern was also observed among individuals who achieved the LDL-C target. However, the low LDL-C/high non-HDL-C subgroup was small; therefore, this estimate should be interpreted with caution. Mechanistically, the discordant phenotype likely reflects a persistent excess of triglyceride-rich and remnant lipoproteins, such that LDL-C appears “at target”, whereas the overall burden of atherogenic apolipoprotein B (apoB)-containing particles remains high [39, 40]. Remnant cholesterol exerts potent atherogenic effects, including efficient arterial wall penetration, uptake by macrophages without prior oxidative modification, and the promotion of low-grade vascular inflammation. Chronic metabolic disturbances that are common in PAD, such as insulin resistance and metabolic syndrome, can further aggravate this profile by reducing lipoprotein lipase activity, promoting hepatic very-low-density lipoprotein overproduction, and impairing remnant clearance, for example through upregulated apolipoprotein C-III, giving rise to the characteristic “atherogenic dyslipidemia” of high triglyceride and small dense low-density lipoprotein particles [40–42]. Consistent with this mechanistic framework, Wadström et al. showed that PAD incidence increased with higher remnant cholesterol levels, and that concomitantly elevated remnant cholesterol explained most of the apparent causal impact of elevated LDL-C on PAD risk, suggesting that remnant cholesterol may represent a key atherogenic cholesterol fraction driving PAD [16, 17]. Thus, in the metabolic and lipid milieu of PAD, LDL-C alone may underestimate the true atherogenic lipid burden, whereas non-HDL-C may more effectively identify the residual cardiovascular risk by capturing a broader spectrum of triglyceride-rich and remnant lipoproteins. Consistent with this, previous statin-treated cohort studies reported higher ASCVD risk linked to higher non-HDL-C [13, 36]. Together, these mechanistic and epidemiological data are consistent with the present findings.

Johannesen et al. reported that lipid discordance was linked to increased all-cause mortality in statin-treated patients [36], whereas this relationship was not observed in the present PAD cohort. This discrepancy may reflect PAD-specific competing risks, including non-atherosclerotic causes such as infections and limb complications, which are strongly influenced by factors beyond lipid burden (e.g., comorbid conditions, frailty, and glycemic control) [43]. All-cause mortality is an etiologically heterogeneous endpoint, and deaths arising through pathways only weakly related to atherogenic lipid exposure may dilute the contribution of lipid-related risk. This dilution may be particularly apparent in small discordant strata, where the estimates are less precise. Consistent with this explanation, the estimates for all-cause mortality were directionally similar to those for cardiovascular death but had wider confidence intervals. These findings suggest that cumulative non-HDL-C may be more closely related to atherosclerosis-driven cardiovascular death than to mortality from all causes. Larger, multi-ethnic PAD cohorts and broader ASCVD cohorts with cause-specific analyses are warranted to determine whether this endpoint-specific pattern is reproducible.

Notably, individuals with higher cumulative non-HDL-C burden were generally younger and had fewer comorbidities (including cardiovascular disease and diabetes), and were less likely to receive statins or other baseline therapies. This apparent paradox likely reflects real-world treatment and selection effects: older, more comorbid patients are more likely to receive intensive treatment and closer monitoring, achieving lower lipid levels despite a higher underlying risk. This “reverse risk factor” pattern has also been described in other high-risk cohorts [44, 45]. During the 2-year exposure window, the proportion of patients receiving statin therapy was higher than that at baseline. Such changes in lipid-lowering treatment are reflected in the estimated cumulative non-HDL-C burden. In clinically higher-risk patients, treatment initiation or intensification may have lowered cumulative non-HDL-C levels and shifted patients toward lower cumulative exposure categories, which tended to attenuate rather than exaggerate the observed association with cardiovascular death. Nevertheless, the higher CV death risk observed with higher cumulative non-HDL-C burden persisted after additional adjustment for changes in statin intensity during the exposure window and in analyses excluding patients with baseline cardiovascular comorbidities, supporting the robustness of the observed association, although residual confounding cannot be excluded.

Clinically, discordant lipid subgroups warrant particular attention. Although individuals with discordant lipid profiles accounted for only approximately 6% of the study cohort, the highest observed proportion of individuals experiencing CV death across these subgroups was 30%. Considering the global prevalence of PAD, this pattern may affect a substantial number of patients globally. Among individuals meeting the prespecified LDL-C-control criteria, those in the highest cumulative non-HDL-C category, in whom the mean non-HDL-C concentration was markedly higher than in the reference group (3.12 vs. 1.55 mmol/L), also experienced higher cardiovascular mortality; however, these values should be interpreted as descriptive summaries rather than actionable cut-offs, and guideline-recommended non-HDL-C targets remain the most practical goals in routine care [5]. As a pragmatic approach, clinicians could approximate the cumulative non-HDL-C burden from the average of several routine measurements, whereas future electronic health record-integrated tools could compute an annualized cumulative burden that accounts for irregular testing intervals, subject to external validation before clinical use. In line with these findings, two large cohorts reported a similar prevalence of discordance (~ 8%–11%) and higher risks of cardiovascular events and mortality [36, 46], supporting further evaluation of non-HDL-C-targeted therapies for residual cardiovascular risk after optimal LDL-C control [47, 48].

In addition, apoB may provide greater precision than non-HDL-C for ASCVD risk because it directly quantifies the number of circulating atherogenic particles, which may be more proximal to the causal pathway than the cholesterol mass alone [36, 49–52]. Non-HDL-C values can be calculated from routine lipid measurements without the need for additional assays, making them highly feasible for routine use, particularly in resource-limited settings. Non-HDL-C is strongly correlated with apoB [53], and previous studies have shown similar risk discrimination between these two markers [54]. In this real-world PAD cohort, apoB was measured in only a small minority of patients and could not be reliably modeled. This represents an important limitation, and future studies with systematic apoB assessments are warranted.

### Strengths and limitations

First, key strengths include the use of a territory-wide, real-world electronic health record database with longitudinal follow-up and comprehensive clinical information, providing a broad basis for assessing lipid exposure and clinical outcomes in routine practice. Second, early cumulative exposure for lipid measures was estimated using serial measurements instead of a single time-point value. This approach reduces the influence of random short-term variations and provides a more stable estimate of sustained lipid burden, which is more relevant to the cumulative atherogenic process underlying cardiovascular risk. By incorporating serial measurements over time, this approach may also reduce regression dilution caused by within-person lipid variability [55, 56]. Third, multiple sensitivity analyses across exposure definitions, lipid measurement requirements, covariate adjustments, baseline comorbidity status, and LDL-C target definitions strengthened the robustness of these findings.

Important limitations should also be considered. First, residual confounding inherent to the observational design cannot be fully excluded, and the findings should be extrapolated cautiously to other ethnic populations and healthcare settings. Second, lipid levels were derived from routine assays in individual hospital laboratories rather than from a central laboratory; thus, some random, non-differential measurement variability is unavoidable and is expected to attenuate associations, despite the repeated measurements used to stabilize cumulative exposure estimates. Third, some LDL-C/non-HDL-C discordant strata included relatively few patients and events, yielding wide confidence intervals; these subgroup findings require cautious interpretation and validation in larger cohorts. Fourth, the strict definition of “well-controlled LDL-C” may have selected patients with more stable lipid control and better adherence, limiting applicability to PAD patients with sustained LDL-C control, although results were consistent using a more relaxed LDL-C definition. Adherence was assessed using prescription-based PDC and may not fully capture real-time medication use; thus, some adherence misclassifications are likely. Fifth, cumulative exposure reflects both the underlying lipid burden and treatment intensity over time and cannot be fully disentangled in this setting; thus, it should be interpreted as a composite measure of the on-treatment lipid burden. Finally, only a small minority of patients in this cohort received non-statin lipid-lowering therapies, which were not modeled in detail; this residual confounding factor may have attenuated the observed associations. Confirmation in multi-ethnic, multicenter prospective cohorts with a broader use of contemporary agents is warranted.

## Conclusion

This study found that, in patients with PAD, higher early cumulative non-HDL-C exposure was associated with a higher cardiovascular death risk, and this association was also observed among individuals with well-controlled LDL-C. In clinical practice, these findings support the incorporation of longitudinal non-HDL-C burden, together with LDL-C levels, into routine lipid follow-up and risk surveillance for patients with PAD. This may help identify residual cardiovascular risk that is not fully captured by LDL-C alone and may provide additional information for strengthening secondary prevention management, including the reassessment of lipid-lowering strategies. By supporting secondary prevention–oriented cardiovascular care, cumulative non-HDL-C assessment may help address the long-term burden of cardiovascular disease in this high-risk population. Future studies should focus on translating the cumulative non-HDL-C assessment into clinical practice by validating actionable cumulative non-HDL-C thresholds, developing electronic health record–based tools for cumulative lipid burden assessment, and testing whether cumulative non-HDL-C-guided secondary prevention improves outcomes in patients with PAD.

## Supplementary Information

Supplementary Material 1.

## Data Availability

The data can be accessed upon reasonable request through the corresponding author.

## Abbreviations

ACEIs: Angiotensin-converting enzyme inhibitors
ANOVA: Analysis of variance
apoB: Apolipoprotein B
ARBs: Angiotensin receptor blockers
ASCVD: Atherosclerotic cardiovascular disease
AUC: Area under the curve
CAD: Coronary artery disease
CDARS: Clinical Data Analysis and Reporting System
CHD: Coronary heart disease
CI: Confidence interval
CKD: Chronic kidney disease
CLI: Critical limb ischemia
CV: Cardiovascular
eGFR: Estimated glomerular filtration rate
HbA1c: Glycated hemoglobin
HDL-C: High-density lipoprotein cholesterol
HR: Hazard ratio
IC: Intermittent claudication
ICD-9-CM: International Classification of Diseases, Ninth Revision, Clinical Modification
ICD-10: International Classification of Diseases, Tenth Revision
IDI: Integrated discrimination improvement
IDL-C: Intermediate-density lipoprotein cholesterol
IQR: Interquartile range
LDL-C: Low-density lipoprotein cholesterol
MICE: Multiple imputation by chained equations
non-HDL-C: Non-high-density lipoprotein cholesterol
NRI: Net reclassification improvement
PAD: Peripheral artery disease
PDC: Proportion of days covered
PCSK9: Proprotein convertase subtilisin/kexin type 9
SD: Standard deviation
SHR: Subdistribution hazard ratio
TC: Total cholesterol
TG: Triglycerides
VLDL-C: Very-low-density lipoprotein cholesterol
