# Supplementary material for: Cumulative non-high-density lipoprotein cholesterol exposure and residual cardiovascular mortality risk in patients with peripheral artery disease
Source: Lipids Health Dis. 2026 Aug 5;25:217. doi: 10.1186/s12944-026-03022-0 (PMC13725944; doi:10.1186/s12944-026-03022-0)
Supplement: Supplementary file 1 [file 12944_2026_3022_MOESM1_ESM.docx]

**Cumulative Non-High-Density Lipoprotein Cholesterol Exposure and Residual Cardiovascular**

**Mortality Risk in Patients with Peripheral Artery Disease**

**Supplementary Material**

Figure S1. Flowchart of the Study Participants.

Figure S2. Measurement Frequency of Non-HDL-C and LDL-C During the 2-Year Exposure Window

Figure S3. Within-Patient Median Intervals Between Non-HDL-C and LDL-C Measurements

Figure S4. Distribution of 2-Year Proportion of Days Covered (PDC) With Lipid-Lowering Therapy

Figure S5. Associations Between Early Cumulative Exposure to LDL-C and Risk of All-Cause and CV Death.

Figure S6A. Kaplan-Meier Survival Curves for CV Death Stratified by Tertiles of Cumulative Non-HDL-C Exposure.

Figure S6B. Kaplan-Meier Survival Curves for All-cause Death Stratified by Tertiles of Cumulative Non-HDL-C Exposure.

Figure S7. Associations Between Baseline LDL-C Levels and Risk of All-Cause and CV Death.

Figure S8. Associations Between Baseline Non-HDL-C Levels and Risk of All-Cause and CV Death.

Figure S9. Hazard Ratios for All-Cause Death Across Concordant and Discordant Categories of Cumulative Non-HDL-C and LDL-C.

Figure S10. Association Between Cumulative Non-HDL-C Exposure and CV Death Among Individuals with Well-Controlled LDL-C.

Figure S11A. Hazard Ratios for All-Cause and CV Death According to Non-HDL-C Categories with 3-Year Early-Stage Exposure.

Figure S11B. Hazard Ratios for All-Cause and CV Death According to LDL-C Categories with 3-Year Early-Stage Exposure.

Figure S12A. Hazard Ratios for All-Cause and CV Death According to non-HDL-C Categories with 1-Year Early-Stage Exposure.

Figure S12B. Hazard Ratios for All-Cause and CV Death According to LDL-C Categories with 1-Year Early-Stage Exposure.

Figure S13A. Hazard Ratios for All-Cause and CV Death According to Non-HDL-C Categories After Excluding Baseline Cardiovascular Comorbidities.

Figure S13B. Hazard Ratios for All-Cause and CV Death According to LDL-C Categories After Excluding Baseline Cardiovascular Comorbidities.

Figure S14. Association Between Cumulative Non-HDL-C and Cardiovascular Death After Adjustment for Statin Intensity Changes.

Figure S15A. Hazard Ratios for CV Death Across Concordant and Discordant Categories of Cumulative Non-HDL-C and LDL-C (≥3 Lipid Measurements).

Figure S15B. Hazard Ratios for All-Cause Death Across Concordant and Discordant Categories of Cumulative Non-HDL-C and LDL-C (≥3 Lipid Measurements).

Figure S16A. Hazard Ratios for CV Death Across Concordant and Discordant Categories of Cumulative Non-HDL-C and LDL-C (Minimally Adjusted Model).

Figure S16B. Hazard Ratios for All-Cause Death Across Concordant and Discordant Categories of Cumulative Non-HDL-C and LDL-C (Minimally Adjusted Model).

Figure S17. Sensitivity analysis of cumulative non-HDL-C and cardiovascular death among patients with LDL-C <1.4 mmol/L at the start of follow-up.

Table S1. ICD-9-CM/ICD-10 Codes for Comorbidities and Outcomes.

Table S2. Daily dose categories used to define low-, moderate- and high-intensity statin therapy

Table S3. Missing data for study covariates

Table S4. Baseline Characteristics of Participants According to Categories of Cumulative Exposure of LDL-C.

Table S5. Baseline use and intensity of lipid-lowering therapy in patients with PAD

Table S6. Lipid-lowering therapy during the 2-year exposure window after PAD diagnosis

Table S7. Absolute event rates for cardiovascular and all-cause death across tertiles of cumulative non-HDL-C and LDL-C (events per 1,000 person-years)

Table S8. Comparison of Model Performance Between Baseline and Cumulative LDL-C and Non-HDL-C Measures for Predicting CV Death.

**Figure S1.** Flowchart of the Study Participants.

**Figure S2.** Measurement Frequency of Non-HDL-C and LDL-C During the 2-Year Exposure Window

**Figure S3.** Within-Patient Median Intervals Between Non-HDL-C and LDL-C Measurements

**Figure S4.** Distribution of 2-Year Proportion of Days Covered (PDC) With Lipid-Lowering Therapy

**Figure S5.** Associations Between Early Cumulative Exposure to LDL-C and Risk of All-Cause and CV Death.

**Note:** The model was adjusted for age, sex, hypertension, diabetes, eGFR, cardiovascular comorbidities, PAD severity, and use of antiplatelet agents, ACEIs/ARBs, and statins. HR hazard ratio, SHR subdistribution hazard ratio, CI confidence interval, eGFR estimated glomerular filtration rate, PAD Peripheral Artery Disease, ACEIs angiotensin-converting enzyme inhibitors, ARBs angiotensin receptor blockers.

**Figure S6A.** Kaplan-Meier Survival Curves for CV Death Stratified by Tertiles of Cumulative Non-HDL-C Exposure.

**Note:** Non-HDL-C Non-high density lipoprotein cholesterol, CE cumulative exposure.

**Figure S6B.** Kaplan-Meier Survival Curves for All-cause Death Stratified by Tertiles of Cumulative Non-HDL-C Exposure.

**Note:** Non-HDL-C Non-high density lipoprotein cholesterol, CE cumulative exposure.

**Figure S7.** Associations Between Baseline LDL-C Levels and Risk of All-Cause and CV Death.

**Note:** The model was adjusted for age, sex, hypertension, diabetes, eGFR, cardiovascular comorbidities, PAD severity, and use of antiplatelet agents, ACEIs/ARBs, and statins. HR hazard ratio, SHR subdistribution hazard ratio, CI confidence interval, eGFR estimated glomerular filtration rate, PAD Peripheral Artery Disease, ACEIs angiotensin-converting enzyme inhibitors, ARBs angiotensin receptor blockers.

**Figure S8.** Associations Between Baseline Non-HDL-C Levels and Risk of All-Cause and CV Death.

**Note:** The model was adjusted for age, sex, hypertension, diabetes, eGFR, cardiovascular comorbidities, PAD severity, and use of antiplatelet agents, ACEIs/ARBs, and statins. HR hazard ratio, SHR subdistribution hazard ratio, CI confidence interval, eGFR estimated glomerular filtration rate, PAD Peripheral Artery Disease, ACEIs angiotensin-converting enzyme inhibitors, ARBs angiotensin receptor blockers.

**Figure S9.** Hazard Ratios for All-Cause Death Across Concordant and Discordant Categories of Cumulative Non-HDL-C and LDL-C.

**Note:** The model was adjusted for age, sex, hypertension, diabetes, eGFR, cardiovascular comorbidities, PAD severity, and use of antiplatelet agents, ACEIs/ARBs, and statins. HR hazard ratio, CI confidence interval, eGFR estimated glomerular filtration rate, PAD Peripheral Artery Disease, ACEIs angiotensin-converting enzyme inhibitors, ARBs angiotensin receptor blockers.

**Figure S10.** Association Between Cumulative Non-HDL-C Exposure and CV Death Among Individuals with Well-Controlled LDL-C.

**Note:** The model was adjusted for age, sex, hypertension, diabetes, eGFR, cardiovascular comorbidities, PAD severity, and use of antiplatelet agents, ACEIs/ARBs, and statins. SHR subdistribution hazard ratio, CI confidence interval, eGFR estimated glomerular filtration rate, PAD Peripheral Artery Disease, ACEIs angiotensin-converting enzyme inhibitors, ARBs angiotensin receptor blockers.

**Figure S11A.** Hazard Ratios for All-Cause and CV Death According to Non-HDL-C Categories with 3-Year Early-Stage Exposure.

**Note:** The model was adjusted for age, sex, hypertension, diabetes, eGFR, cardiovascular comorbidities, PAD severity, and use of antiplatelet agents, ACEIs/ARBs, and statins. HR hazard ratio, SHR subdistribution hazard ratio, CI confidence interval, eGFR estimated glomerular filtration rate, PAD Peripheral Artery Disease, ACEIs angiotensin-converting enzyme inhibitors, ARBs angiotensin receptor blockers.

**Figure S11B.** Hazard Ratios for All-Cause and CV Death According to LDL-C Categories with 3-Year Early-Stage Exposure.

**Note:** The model was adjusted for age, sex, hypertension, diabetes, eGFR, cardiovascular comorbidities, PAD severity, and use of antiplatelet agents, ACEIs/ARBs, and statins. HR hazard ratio, SHR subdistribution hazard ratio, CI confidence interval, eGFR estimated glomerular filtration rate, PAD Peripheral Artery Disease, ACEIs angiotensin-converting enzyme inhibitors, ARBs angiotensin receptor blockers.

**Figure S12A.** Hazard Ratios for All-Cause and CV Death According to Non-HDL-C Categories with 1-Year Early-Stage Exposure.

**Note:** The model was adjusted for age, sex, hypertension, diabetes, eGFR, cardiovascular comorbidities, PAD severity, and use of antiplatelet agents, ACEIs/ARBs, and statins. HR hazard ratio, SHR subdistribution hazard ratio, CI confidence interval, eGFR estimated glomerular filtration rate, PAD Peripheral Artery Disease, ACEIs angiotensin-converting enzyme inhibitors, ARBs angiotensin receptor blockers.

**Figure S12B.** Hazard Ratios for All-Cause and CV Death According to LDL-C Categories with 1-Year Early-Stage Exposure.

**Note:** The model was adjusted for age, sex, hypertension, diabetes, eGFR, cardiovascular comorbidities, PAD severity, and use of antiplatelet agents, ACEIs/ARBs, and statins. HR hazard ratio, SHR subdistribution hazard ratio, CI confidence interval, eGFR estimated glomerular filtration rate, PAD Peripheral Artery Disease, ACEIs angiotensin-converting enzyme inhibitors, ARBs angiotensin receptor blockers.

**Figure S13A.** Hazard Ratios for All-Cause and CV Death According to Non-HDL-C Categories After Excluding Baseline Cardiovascular Comorbidities.

**Note:** The model was adjusted for age, sex, hypertension, diabetes, eGFR, PAD severity, and use of antiplatelet agents, ACEIs/ARBs, and statins. HR hazard ratio, SHR subdistribution hazard ratio, CI confidence interval, eGFR estimated glomerular filtration rate, PAD Peripheral Artery Disease, ACEIs angiotensin-converting enzyme inhibitors, ARBs angiotensin receptor blockers.

**Figure S13B.** Hazard Ratios for All-Cause and CV Death According to LDL-C Categories After Excluding Baseline Cardiovascular Comorbidities.

**Note:** The model was adjusted for age, sex, hypertension, diabetes, eGFR, PAD severity, and use of antiplatelet agents, ACEIs/ARBs, and statins. HR hazard ratio, SHR subdistribution hazard ratio, CI confidence interval, eGFR estimated glomerular filtration rate, PAD Peripheral Artery Disease, ACEIs angiotensin-converting enzyme inhibitors, ARBs angiotensin receptor blockers.

**Figure S14. Association Between Cumulative Non-HDL-C and Cardiovascular Death After Adjustment for Statin Intensity Changes**

**Note:** Cumulative non-HDL-C exposure was categorized into tertiles, with Tertile 1 as the reference. Statin intensity was assessed at baseline and at the end of the 2-year exposure assessment period and classified as no statin, low intensity, moderate intensity, or high intensity. Changes in statin treatment were categorized as no statin use at either assessment, initiation or intensification, discontinuation or de-intensification, or unchanged intensity. The model was adjusted for age, sex, hypertension, diabetes, eGFR, cardiovascular comorbidities, PAD severity, and use of antiplatelet agents, ACEIs/ARBs, and statin intensity changes. SHR subdistribution hazard ratio CI confidence interval, eGFR estimated glomerular filtration rate, PAD peripheral artery disease, ACEIs angiotensin-converting enzyme inhibitors, ARBs angiotensin receptor blockers.

**Figure S15A.** Hazard Ratios for CV Death Across Concordant and Discordant Categories of Cumulative Non-HDL-C and LDL-C (≥3 Lipid Measurements).

**Note:** The model was adjusted for age, sex, hypertension, diabetes, eGFR, cardiovascular comorbidities, PAD severity, and use of antiplatelet agents, ACEIs/ARBs, and statins. SHR subdistribution hazard ratio, CI confidence interval, eGFR estimated glomerular filtration rate, PAD Peripheral Artery Disease, ACEIs angiotensin-converting enzyme inhibitors, ARBs angiotensin receptor blockers.

**Figure S15B.** Hazard Ratios for All-Cause Death Across Concordant and Discordant Categories of Cumulative Non-HDL-C and LDL-C (≥3 Lipid Measurements).

**Note:** The model was adjusted for age, sex, hypertension, diabetes, eGFR, cardiovascular comorbidities, PAD severity, and use of antiplatelet agents, ACEIs/ARBs, and statins. HR hazard ratio, CI confidence interval, eGFR estimated glomerular filtration rate, PAD Peripheral Artery Disease, ACEIs angiotensin-converting enzyme inhibitors, ARBs angiotensin receptor blockers.

**Figure S16A.** Hazard Ratios for CV Death Across Concordant and Discordant Categories of Cumulative Non-HDL-C and LDL-C (Minimally Adjusted Model).

**Note:** The model was adjusted for age, sex, diabetes, PAD severity, cardiovascular comorbidities, statin use. SHR subdistribution hazard ratio, CI confidence interval, PAD Peripheral Artery Disease.

**Figure S16B.** Hazard Ratios for All-Cause Death Across Concordant and Discordant Categories of Cumulative Non-HDL-C and LDL-C (Minimally Adjusted Model).

**Note:** The model was adjusted for age, sex, diabetes, PAD severity, cardiovascular comorbidities, statin use. HR hazard ratio, CI confidence interval, PAD Peripheral Artery Disease.

**Figure S17.** Sensitivity analysis of cumulative non-HDL-C and cardiovascular death among patients with LDL-C <1.4 mmol/L at the start of follow-up.

**Note:** The model was adjusted for age, sex, hypertension, diabetes, eGFR, cardiovascular comorbidities, PAD severity, and use of antiplatelet agents, ACEIs/ARBs, and statins. SHR subdistribution hazard ratio, CI confidence interval, eGFR estimated glomerular filtration rate, PAD Peripheral Artery Disease, ACEIs angiotensin-converting enzyme inhibitors, ARBs angiotensin receptor blockers.

**Table S1.** ICD-9-CM/ICD-10 Codes for Comorbidities and Outcomes.

| **Disease** | **ICD-9-CM** |
| --- | --- |
| PAD | 440.20, 440.21, 440.22, 440.23, 440.24, 440.29, 440.4, 440.8, 440.9, 443, 443.1, 443.2, 443.8, 443.81, 443.82, 443.89, 443.9 |
| Hypertension | 401, 402, 405, 403, 404, 402.91, 404.91, 404.93 |
| Coronary artery disease | 410, 411, 412, 413, 414, 429.79 |
| Stroke | 433, 434, 435, 436, 437, 438, 430, 431, 432 |
| Congestive Heart Failure | 398.91, 402.91, 404.91, 404.9, 425, 428 |
| Arrhythmias | 426, 427.0, 427.1, 427.2, 427.4, 427.6, 427.8, 427.9 |
| Chronic kidney disease | 250.4, 403, 404, 581, 582, 583, 585, 586, 588, 402.91, 404.91, 404.93 |
| Diabetes | 250.00 |
| Intermittent claudication | 440.21, 443.9:6 |
| Critical limb ischemia | 440.22, 440.23, 440.24, 707.9:4 |
| **Outcomes** | **ICD-10-Code** |
| Cardiovascular related death | I00-I09, I11, I13, I20-I51, I60-I69 |

**Table S2.** Daily dose categories used to define low-, moderate- and high-intensity statin therapy

| **Statin** | **Low-intensity dosage (LDL-C reduction <30%)** | **Moderate-intensity dosage (LDL-C reduction 30% to <50%)** | **High-intensity dosage (LDL-C reduction ≥50%)** |
| --- | --- | --- | --- |
| **Atorvastatin** | NA | 10–20 mg | 40–80 mg |
| **Fluvastatin** | 20–40 mg | 40 mg BID; XL 80 mg | NA |
| **Lovastatin** | 20 mg | 40–80 mg | NA |
| **Pitavastatin** | NA | 1–4 mg | NA |
| **Pravastatin** | 10–20 mg | 40–80 mg | NA |
| **Rosuvastatin** | NA | 5–10 mg | 20–40 mg |
| **Simvastatin** | 10 mg | 20–40 mg | NA |

**Note:** BID, twice daily; XL, extended release; NA, not applicable.

**Table S3.** Missing data for study covariates

| **Covariates** | **Missing rate** |
| --- | --- |
| **Age** | 0% |
| **Sex** | 0% |
| **Comorbidities**  (hypertension, cardiovascular comorbidities, PAD severity,  diabetes) | 0% |
| **Medications**  (antiplatelet agents, ACEI/ARB, statins) | 0% |
| **eGFR** | 0.16% (only 6) |

**Table S4.** Baseline Characteristics of Participants According to Categories of Cumulative Exposure of LDL-C.

| Characteristics | Tertile 1  N = 1,283 | Tertile 2  N = 1,282 | Tertile 3  N = 1,282 | Total  N = 3,847 | P value |
| --- | --- | --- | --- | --- | --- |
| LDL-C (Cumulative Exposure), mmol/L | 1.73 [1.54, 1.88] | 2.25 [2.13, 2.38] | 2.89 [2.68, 3.22] | 2.25 [1.88, 2.68] | <0.001 |
| LDL-C (Baseline), mmol/L | 1.67 [1.38, 1.99] | 2.33 [2.01, 2.68] | 3.22 [2.69, 3.82] | 2.32 [1.79, 3.05] | <0.001 |
| Demographic |  |  |  |  |  |
| Male Gender, % | 850 (66.3%) | 804 (62.7%) | 768 (59.9%) | 2422 (63.0%) | 0.004 |
| Age, years | 71.15 ± 10.74 | 70.34 ± 11.24 | 68.59 ± 11.77 | 70.03 ± 11.30 | <0.001 |
| Comorbidities |  |  |  |  |  |
| Hypertension, % | 805 (62.7%) | 725 (56.6%) | 629 (49.1%) | 2159 (56.1%) | <0.001 |
| Cardiovascular disease, % | 854 (66.6%) | 694 (54.1%) | 514 (40.1%) | 2062 (53.6%) | <0.001 |
| CKD, % | 304 (23.7%) | 286 (22.3%) | 241 (18.8%) | 831 (21.6%) | 0.008 |
| Diabetes, % | 882 (68.7%) | 796 (62.1%) | 700 (54.6%) | 2378 (61.8%) | <0.001 |
| Laboratory |  |  |  |  |  |
| Total Cholesterol, mmol/L | 3.48 [3.05, 3.95] | 4.20 [3.77, 4.70] | 5.17 [4.58, 5.85] | 4.20 [3.57, 5.00] | <0.001 |
| High-density lipoprotein, mmol/L | 1.10 [0.88, 1.36] | 1.11 [0.93, 1.37] | 1.15 [0.96, 1.40] | 1.12 [0.92, 1.38] | <0.001 |
| Triglycerides, mmol/L | 1.21 [0.88, 1.69] | 1.30 [0.95, 1.83] | 1.40 [1.02, 1.93] | 1.30 [0.95, 1.82] | <0.001 |
| HbA1c, % | 6.90 [6.13, 7.80] | 6.96 [6.20, 8.10] | 6.90 [6.20, 8.00] | 6.90 [6.20, 7.92] | 0.196 |
| eGFR, mL/min/1.73 m² | 54.94 [31.15, 77.19] | 58.50 [33.14, 82.06] | 60.75 [35.14, 83.91] | 58.19 [33.17, 81.10] | <0.001 |
| Medication |  |  |  |  |  |
| ACEI/ARBs | 868 (67.7%) | 810 (63.2%) | 655 (51.1%) | 2333 (60.6%) | <0.001 |
| Beta-blockers | 654 (51.0%) | 503 (39.2%) | 447 (34.9%) | 1604 (41.7%) | <0.001 |
| Antiplatelet | 1032 (80.4%) | 961 (75.0%) | 852 (66.5%) | 2845 (74.0%) | <0.001 |
| Antidiabetic drugs | 536 (41.8%) | 447 (34.9%) | 362 (28.2%) | 1345 (35.0%) | <0.001 |
| Statin | 1071 (83.5%) | 852 (66.5%) | 637 (49.7%) | 2560 (66.5%) | <0.001 |

**Notes:** Values are presented as mean ± SD, median (IQR), or n (%), as appropriate. Analyses were based on cumulative LDL-C levels during the early exposure period. LDL-C low-density lipoprotein cholesterol, CKD chronic kidney disease, HbA1c glycated hemoglobin, eGFR estimated glomerular filtration rate, ACEIs angiotensin-converting enzyme inhibitors, ARBs angiotensin receptor blockers.

**Table S5.** Baseline use and intensity of lipid-lowering therapy in patients with PAD

| **Lipid type** | **N (%)** |
| --- | --- |
| **Patients with STATIN** | 2,560 (66.5%) |
| High | 211 (5.5%) |
| Moderate | 1276 (33.2%) |
| Low | 810 (21.1%) |
| NA | 263 (6.8%) |
| **Monotherapy** |  |
| Statin | 2,441 (63.5%) |
| Fibrate | 68 (1.8%) |
| Ezetimibe | 2 (0.1%) |
| PCSK9i | 6 (0.2%) |
| Other Lipid Lowering | 3 (0.1%) |
| **Combination therapy** |  |
| Statin + Fibrate | 74 (1.9%) |
| Statin + Ezetimibe | 37 (1.0%) |
| Statin + PCSK9i | 3 (0.1%) |
| Statin + Other Lipid Lowering | 5 (0.1%) |
| **No lipid** | 1,208 (31.4%) |

**Note:** Baseline lipid-lowering therapy was assessed from prescription records around the time of PAD diagnosis (from 6 months before to 1 week after the index PAD diagnosis). Monotherapy denotes prescriptions for a single lipid-lowering agent only, whereas combination therapy denotes prescriptions for ≥2 different lipid-lowering agents within any 3-month period. Statin intensity (high, moderate, low) was classified according to Table S2 based on the statin regimen in the prescription closest to the index PAD diagnosis. “Other lipid-lowering” includes agents other than statins, fibrates, ezetimibe, and PCSK9 inhibitors. PCSK9i, proprotein convertase subtilisin/kexin type 9 inhibitor; NA, not available.

**Table S6.** Lipid-lowering therapy during the 2-year exposure window after PAD diagnosis

| **Lipid type** | **N (%)** |
| --- | --- |
| Patients with STATIN | 3,132 (81.4%) |
| Statin without high-intensity | 2,687 (69.8%) |
| Ever high-intensity statin | 445 (11.6%) |
| Patients with other lipid-lowering medications | 255 (6.6%) |
| **Monotherapy** |  |
| Statin | 2,938 (76.4%) |
| Fibrate | 107 (2.8%) |
| Ezetimibe | 17 (0.4%) |
| PCSK9i | 7 (0.2%) |
| Other Lipid Lowering | 4 (0.1%) |
| **Combination therapy** |  |
| Statin + Fibrate | 99 (2.6%) |
| Statin + Ezetimibe | 72 (1.9%) |
| Statin + PCSK9i | 9 (0.2%) |
| Statin + Other Lipid Lowering | 11 (0.3%) |
| Other combination | 5 (0.1%) |
| **No lipid** | 654 (17.0%) |

**Note:** Lipid-lowering therapy during the 2-year exposure window was based on prescriptions issued within 2 years after the index PAD diagnosis. Patients were considered to be receiving a given agent if at least one prescription for that agent was issued during this period. Monotherapy denotes prescriptions for a single lipid-lowering agent only, whereas combination therapy denotes prescriptions for ≥2 different lipid-lowering agents within any 3-month period. “Other lipid-lowering” includes agents other than statins, fibrates, ezetimibe, and PCSK9 inhibitors. “No lipid” indicates that no lipid-lowering prescriptions were recorded during the 2-year exposure window. PCSK9i, proprotein convertase subtilisin/kexin type 9 inhibitor.

**Table S7.** Absolute event rates for cardiovascular and all-cause death across tertiles of cumulative non-HDL-C and LDL-C (events per 1,000 person-years)

| **Outcome** | **Lipid cumulative exposure** | **T1** | **T2** | **T3** |
| --- | --- | --- | --- | --- |
| **Cardiovascular death** | Non-HDL-C | 25.0 | 25.8 | 28.8 |
|  | LDL-C | 25.6 | 26.8 | 27.5 |
| **All-cause death** | Non-HDL-C | 97.5 | 101.1 | 101.2 |
|  | LDL-C | 95.1 | 101.6 | 103.4 |

**Note:** Values are absolute event rates (events per 1,000 person-years). T1–T3, tertiles of cumulative lipid exposure.

**Table S8.** Comparison of Model Performance Between Baseline and Cumulative LDL-C and Non-HDL-C Measures for Predicting CV Death

| **Model** | **C-index(95%CI)** | **Δ-C index (95% CI)** | **IDI (95% CI)** | **NRI (95% CI)** |
| --- | --- | --- | --- | --- |
| **Confounders + baseline LDL-C** | 0.6647(0.6435-0.6859) | Ref | Ref | Ref |
| **Confounders + Cumulative LDL-C** | 0.6662(0.6452-0.6872) | 0.0015 (0.0002-0.0027) | 0.0026 (0.0017-0.0034) | 22.8% (16.5%-29.2%) |
| **Confounders + baseline non-HDL-C** | 0.6650(0.6438-0.6863) | Ref | Ref | Ref |
| **Confounders + Cumulative non-HDL-C** | 0.6677(0.6466-0.6887) | 0.0027 (0.0007-0.0046) | 0.0024 (0.0014-0.0033) | 15.8% (9.6%-21.9%) |
| **Confounders + Cumulative LDL-C** | 0.6662(0.6452-0.6872) | Ref | Ref | Ref |
| **Confounders + Cumulative non-HDL-C** | 0.6677(0.6466-0.6887) | 0.0015 (0.0003-0.0028) | 0.0017 (0.0010-0.0024) | 13.3% (6.8%-19.8%) |

**Note:** C-index, IDI, and NRI with 95% CIs were used to compare model performance. Confounders include age, sex, hypertension, diabetes, eGFR, cardiovascular comorbidities, PAD severity, and use of antiplatelets, ACEIs/ARBs, and statins. C-index concordance index, IDI integrated discrimination improvement, NRI net reclassification improvement, CI confidence interval, eGFR estimated glomerular filtration rate, PAD peripheral artery disease, ACEIs angiotensin-converting enzyme inhibitors, ARBs angiotensin receptor blockers.
